# Equol, a Dietary Daidzein Gut Metabolite Attenuates Microglial Activation and Potentiates Neuroprotection In Vitro

**DOI:** 10.3390/nu9030207

**Published:** 2017-02-27

**Authors:** Lalita Subedi, Eunhee Ji, Dongyun Shin, Jongsik Jin, Joo Hong Yeo, Sun Yeou Kim

**Affiliations:** 1 College of Pharmacy, Gachon University, #191, Hambakmoero, Yeonsu-gu, Incheon 21936, Korea; subedilali@gmail.com (L.S.); ehji@gachon.ac.kr (E.J.); dyshin@gachon.ac.kr (D.S.); 2 Department Oriental Medicine Resources, College of Environmental & Bioresources Sciences, Chonbuk National University, Jeonju City 54896, Korea; jongsik.jin@jbnu.ac.kr; 3 National Institute of Biological Resources, Environmental Research Complex, 42 Hwangyeong-ro, Seo-gu, Incheon 22689, Korea; naasyeo@korea.kr; 4 Gachon Medical Research Institute, Gil Medical Center, Inchon 21565, Korea; 5 Gachon Institute of Pharmaceutical Science, Gachon University; #191 Hambakmoe-ro, Yeonsu-gu, Incheon 21565, Korea

**Keywords:** Equol, phytoestrogen, neuroinflammation, apoptosis, neuroprotection

## Abstract

Estrogen deficiency has been well characterized in inflammatory disorders including neuroinflammation. Daidzein, a dietary alternative phytoestrogen found in soy (*Glycine max*) as primary isoflavones, possess anti-inflammatory activity, but the effect of its active metabolite Equol (7-hydroxy-3-(4′-hydroxyphenyl)-chroman) has not been well established. In this study, we investigated the anti-neuroinflammatory and neuroprotective effect of Equol in vitro. To evaluate the potential effects of Equol, three major types of central nervous system (CNS) cells, including microglia (BV-2), astrocytes (C6), and neurons (N2a), were used. Effects of Equol on the expression of inducible nitric oxide synthase (iNOS), cyclooxygenase (COX-2), Mitogen activated protein kinase (MAPK) signaling proteins, and apoptosis-related proteins were measured by western blot analysis. Equol inhibited the lipopolysaccharide (LPS)-induced TLR4 activation, MAPK activation, NF-kB-mediated transcription of inflammatory mediators, production of nitric oxide (NO), release of prostaglandin E2 (PGE-2), secretion of tumor necrosis factor-α (TNF-α) and interleukin 6 (IL-6), in Lipopolysaccharide (LPS)-activated murine microglia cells. Additionally, Equol protects neurons from neuroinflammatory injury mediated by LPS-activated microglia through downregulation of neuronal apoptosis, increased neurite outgrowth in N2a cell and neurotrophins like nerve growth factor (NGF) production through astrocytes further supporting its neuroprotective potential. These findings provide novel insight into the anti-neuroinflammatory effects of Equol on microglial cells, which may have clinical significance in cases of neurodegeneration.

## 1. Introduction

Nutraceuticals may be foods or nutrients that provide health benefits, including the prevention or treatment of a disease [1]. Nutraceuticals and their active metabolites have been reported to possess a variety of biological activities [2]. Fewer adverse effects, easy availability and promising effects of nutraceuticals and phytochemicals are advantages that drive the development of safe, effective, and economic new drugs for better health [3].

Among those dietary foods that have pharmacologically active metabolites, soybean (*Glycine max* L.), which is commonly consumed in traditional Asian food, is a major source of oil and protein in the human and animal diet [4]. It is one of the natural substitutes for the treatment of a variety of hormone-dependent and independent disorders in humans [5]. Biologically active soy phytoestrogens and their potential effects against breast, prostate, and colon cancer, obesity, cardiovascular disease, Alzheimer’s disease, and osteoporosis have made them the subject of research for a long time [5,6]. Isoflavones are phytoestrogens with potent estrogenic activity; Genistein, Daidzein, and glycitein are the most active of the isoflavones found in soybeans [7,8]. Soy metabolites, namely isoflavones, unsaturated fatty acids, and saponins, possess great potential for human health due to their antioxidant and anti-carcinogenic activity [9]. Soy isoflavones, or phytoestrogens from soy, have been shown to be strongly associated with anticancer activity and inhibition of the epidermal growth factor receptor tyrosine kinase [10]. Furthermore, Schreihofer et al. reported that pretreatment with soy phytoestrogens might mimic in vitro neuroprotective effects [11]. In addition, Ma W et al. suggested that Genistein may possess neuroprotective properties through its anti-inflammatory activity [12].

Glycosides, like Daidzin (Daidzein glycoside) and Genistin (Genistein glycoside) [13], are biologically active candidates for a variety of human ailments [8,14,15]. Not all but about 20%–35% of Western and 50%–55% Asian adult populations are Equol producers [16]. They can metabolize Daidzein by gut microflora to an estrogenic metabolite called Equol [7-hydroxy-3-(4′-hydroxyphenyl)-chroman], which exhibits biological properties that exceed those of its precursor [17,18,19,20]. Equol is similar in structure to the human female hormone, 17-β-estradiol [21]. It can bind to both alpha and beta estrogen receptors, where it mimics the action of estrogens on target organs, thereby exerting health benefits when used in some hormone-dependent diseases [21,22]. Equol displays strong antioxidant properties, as well as the ability to regulate cell cycles [23,24]. Interestingly, soybean was reported to act as an acetyl cholinesterase inhibitor and was found to improve memory in rodents using different maze models. In addition, the neuroprotective effect of Equol in transient focal cerebral ischemia was reported [25,26].

Oxidative stress and neuroinflammation are believed to play key roles in nigrostriatal dopaminergic neuron demise [27]. Neuroprotective effects of estrogens have been widely reported in a number of neuronal cells [28]. Besides having potential as various disease treatments, estrogen has many acute and long-term adverse effects, including headaches and migraines [29]. Estrogen therapy can be dangerous to those who are prone to blood clotting and it can increase the risk of uterine, breast and endometrial cancer, liver complications, gallstones and cholecystectomy [30]. For this reason, research into an estrogen-like phytoestrogen without toxicities is ongoing. Soy and soy products such as Genistein and Daidzein have previously been reported to possess the ability to treat various CNS disorders including depression, Alzheimer’s, epilepsy, dementia etc. Moreover, Daidzein and its metabolites are the major interest of research these days for their anti-neuroinflammatory efficacy [31]. As Equol and *O*-desmethyl angolensin (ODMA) are the major intestinal metabolites of Daidzein, ODMA is reported to have little potential to bind with the estrogen receptors (ERs) in comparison to that of Daidzein and Equol [32,33]. Estrogen receptor (ER) beta agonists have been demonstrated to possess anti-inflammatory properties in inflammatory disease models [34]. Moreover, Equol, being a strong ER-β agonist, can not only provide the positive therapeutic value as hormonal therapy but also help to control various neurodegenerative disorders [34]. Hence, exploring the biological activity of Daidzin and its metabolite especially Equol could be of great interest for neuroinflammatory disorders.

Microglial cells, the immune resident cells of the brain, are principally responsible for immune defense in the CNS. However, under pathological conditions, microglia cells are over-activated and produce a variety of proinflammatory mediators, including NO. For the in vitro experiment we used Lipopolysaccharide (LPS) as a neuroinflammation inducer that is responsible for neurodegeneration. LPS is a bacterial endotoxin that can trigger the inflammation via microglia and astrocyte activation, followed by increased production of COX-2, iNOS, NO and proinflammatory cytokine such as TNF-α and IL-6, which ultimately cause damage to DA neuron that causes various neuroinflammatory and neurodegenerative disease. [35,36]. In this study, we evaluated Equol and its related compounds for inhibitory effects on LPS-activated NO production in BV-2 cells. Together with the result we further hypothesized that Equol, a gut metabolite of Daidzein, might have more anti-neuroinflammatory potential than Daidzein. The detailed mechanism has not been well established and its neuroprotective role in vitro is not yet determined. Therefore, in this study, we are to determine whether Equol attenuates proinflammatory responses in LPS-stimulated BV-2 microglia and attempted to clarify the possible mechanisms of action. LPS is an endotoxin found in the cell wall of gram-negative bacteria and it is extensively used to induce the neuroinflammation in experimental models both in in vivo and in vitro [37]. In this study we used LPS obtained from the *Escherichia coli* strain to activate the BV2 microglial cells.

## 2. Experimental Section

### 2.1. Reagents

Chemicals used in cell culture experiments including Dulbecco’s modified Eagle medium (DMEM), fetal bovine serum (FBS), and penicillin-streptomycin were obtained from Invitrogen (Carlsbad, CA, USA). LPS, *N*-monomethyl-l-arginine (NMMA), and (R, S)-Equol were purchased from LC Laboratories (Cat. No.: E-5880). Enzyme link immune sorbent assay (ELISA) development kit, TNF-α, IL-6, PGE-2, and NGF were purchased from R&D Systems (Minneapolis, MN, USA). Primary and secondary antibodies for iNOS, COX-2, pERK, ERK, pJNK, JNK, pp38, p38, Bax, Bcl-2, cleaved caspase-3, and tubulin were purchased from Cell Signaling (Beverly, MA, USA). All other chemicals and reagents were purchased from Sigma Chemical (St. Louis, MO, USA) unless otherwise stated.

### 2.2. Cell Culture

Three different cell lines microglia (BV2), astrocytes (C6) and Neuroblastoma (N2a) were used to study the anti-neuroinflammatory and neuroprotective effect of Equol. Unlike other studies that used particular cell types for the study of the anti-neuroinflammatory efficacy of different targets, the use of three different cell lines in this study represents the complete CNS environment. The murine BV2 cell lines were kindly provided as gift samples from E. Choi at Korea University (Seoul, South Korea). We purchased the C6 glioma cells from the Korean Cell Line Bank (Seoul, Korea), whereas the N2a cells were obtained from American Type Culture Collection (Manassas, VA, USA). All cell culture and maintenance procedure was done as described previously [38].

### 2.3. Cell Viability Assay

3-(4,5-dimethylthiazol-2-yl)-2,5-diphenyl-tetrazolium bromide (MTT) assay was used to determine the cell viability of BV2 cells 24 h after the exposure with LPS in the presence or absence of different concentrations of Equol as described previously [38] with some modification. Supernatant or medium was removed from the treated plate and MTT solution was added in the cell and it was incubated for an hour. After incubation the solution was also removed and DMSO was added to convert blue stained cells (MTT) to Formazan.

### 2.4. Nitric Oxide (NO) and Proinflammatory Cytokine Measurement

The inhibitory effect of Equol on LPS-stimulated NO synthesis and proinflammatory cytokine release was studied using BV-2 microglial cells. BV-2 cells were plated into a 96well plate (4 × 10^4^ cells/well) and treated with 100 ng/mL LPS [37], in the presence or absence of different concentrations of Equol, for 24 h with NG-mono-methyl-l-arginine (L-NMMA), a well-known nitric oxide synthase (NOS) inhibitor [39] as a positive control. Nitrite level was measured in the culture media using Gries reagent as described previously [38] with some modification. Equal amount of treated cells supernatant or conditioned medium, i.e., 50 µL was added to 50 µL of Gries reagent and the colorimetric changes were measured at 540 nm wavelength in the microplate reader taking Nano2 as a standard.

### 2.5. Measurement of PGE-2, TNF-α, and IL-6 Production

The level of PGE-2, TNF-α, and IL-6 production were measured in culture medium of LPS- stimulated BV2 cells in the presence or absence of Equol at different concentrations. Following 24 h of incubation, the levels of PGE-2, TNF-α, and IL-6 was measured. PGE-2 was measured using a competitive ELISA (Cayman Chemical, Ann Arbor, MI, USA) as described previously [38].

### 2.6. NF-κB Assay

Nuclear extracts from the LPS-stimulated microglia were prepared using a Nuclear Extract Kit (Active Motif, Carlsbad, CA, USA) according to the manufacturer’s protocol. Protein levels of NF-κB, IκB and p-IκB were evaluated by western blot analysis.

### 2.7. Western Blot Analysis

BV2 cells were seeded in a 6-well plate at the density of 6 × 10^5^ cells/well and treated with 5, 10, 20 μM and activated with LPS (100 ng/mL) and incubated for 24 h. Cells were washed with ice-cold PBS and collected using a scraper. Collected cells were lysed with lysis buffer (50 mM Tris-HCl, pH 8.0, 0.1% SDS, 150 mM NaCl, 1% NP-40, 0.02% sodium azide, 0.5% sodium deoxycholate, 100 µg/mL PMSF, 1 g/mL approtinin) and the proteins obtained were estimated using Bradford’s assay in order to get 30 μg of it from each group. Equol protein was loaded and separated by 10% SDS-PAGE gel electrophoresis, transferred to nitrocellulose membranes, blocked with 5% skim-milk and incubated overnight with primary antibodies against tubulin (Sigma-Aldrich, St. Louis, MO, USA, Cat. No.: T5168), iNOS (Bioscience Cat. No.: 610333, COX-2 (Santa Cruz, CA, USA Cat. No.: sc-1745), ERK (Cell Signaling, Danvers, MA, USA Cat. No.: 9107s), pERK (Cell Signaling Cat. No.: 5013s), JNK (Cell Signaling Cat. No.: 9252s), pJNK (Cell Signaling Cat. No.: 4671s), p38 (Cell Signaling Cat. No.: 8690s), pP38 Cell Signaling Cat. No.: 9211s), cleaved caspase-3 (Cell Signaling Cat. No.: 9661s), Bax (Santa Cruz Cat. No.: Sc-493), and Bcl-2 (Santa Cruz, CA, USA, Cat. No.: Sc492) at 4°C. Membranes were then incubated with respective horseradish peroxidase-conjugated secondary antibodies and protein bands were visualized using enhanced chemiluminescence reagent using the Chemi DocXRS+ imaging system (Bio-Rad, Hercules, CA, USA). Densitometry analysis of the bands was done by using Image Master™ 2D Elite software (version 3.1, Amersham Pharmacia Biotech, Buckinghamshire, UK) as described previously [38].

### 2.8. Neurite Outgrowth Assay

For neurite outgrowth assay, N2a cells were seeded onto 12 well plates at a density of 6 × 10^5^ cells/well and treated with Equol for 24 h [38]. The extent of neurite outgrowth was determined by measuring neurite lengths of N2a cells using IncuCyte imaging system from zero to 24 h of treatment in each 2 h interval (Essen Instruments, Ann Arbor, MI, USA) as previously reported [38].

### 2.9. NGF Assay

C6 glioma cells were seeded onto 24 well plates at a density of 1 × 10^5^ cells/well and treated with serum-free DMEM and the different concentrations of Equol for 24 h. Cultured media was used to measure the amount of NGF released into the medium using an ELISA development kit as previously described [38].

### 2.10. Statistical Analysis

All experimental data are represented as mean ± SEM. Level of significance were determined using one-way ANOVA, followed by the Newman-Keuls post hoc test, using GraphPad Prism 5 (GraphPad Software Inc., La Jolla, CA, USA). *p* < 0.05 was set as statistically significant. For the reproducibility of the results, each experiment was performed in triplicate.

## 3. Results

### 3.1. Effect of Daidzein and Its Derivatives on NO Production in LPS-Stimulated BV-2 Cells

**Figure 1:**
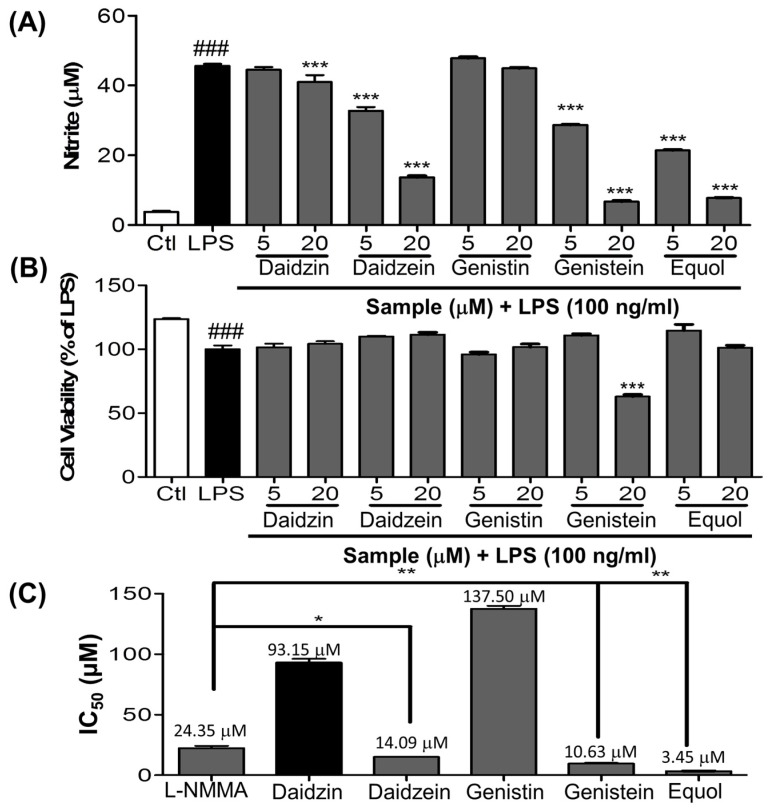
Effects of Equol and its derivatives on NO production in LPS-stimulated BV-2 cells. BV-2 cells were pretreated with 5, and 20 μM of Equol and its derivatives for 30 min and stimulated with LPS (100 ng/mL) for 24 h. (**A**) NO Production Assay; (**B**) Cell viability on BV-2 microglia was determined using an MTT assay; (**C**) IC_50_ value graph for Equol and its derivatives. All data are presented as the mean ± SEM of three independent experiments. * *p* < 0.05, ** *p* < 0.01 and *** *p* < 0.001 vs. LPS-treated group and ^###^ *p* < 0.001 vs. untreated control group.

An increase in NO production has been believed to be a biomarker for inflammation, especially neuroinflammation and related disease conditions. We performed the screening of Daidzin and its derivative including Equol for their inhibitory activity on NO production in LPS-activated BV2 cells. LPS increased the NO production from 3.77 ± 0.25 in the control to 45.61 ± 0.52 in the LPS treated group. However, 20 µM of Daidzin, Daidzein, Genistin, Genistein and Equol reduced the NO production to 40.97 ± 2.00, 13.72 ± 0.38, 44.88 ± 0.25, 6.74 ± 0.24 and 7.73 ± 0.11 µM, respectively, in LPS stimulated BV2 cells. Genistein showed better activity at higher concentration, but at the same time it showed almost 40% cellular toxicity to BV2 cells, and because of this reason the NO production value is lower than Equol. Also, among the tested compounds, Equol showed the most potent inhibitory effect on NO production in LPS-stimulated BV-2 cells as shown in Figure 1A,B. Therefore, Equol is the most potent inhibitor of NO production among all derivatives. We found that Daidzein and related compounds showed promising effects on inhibition of NO production, providing the clue for the anti-inflammatory activity of Daidzein and its related compounds among them, Equol showed the most potent inhibitory effect on NO production in LPS-stimulated BV-2 cells as shown in Figure 1A,B.

### 3.2. Dose Response Effect of Equol on NO Production, iNOS and COX-2 Expression and TLR4 Inactivation in LPS-Stimulated BV-2 Cells

**Figure 2:**
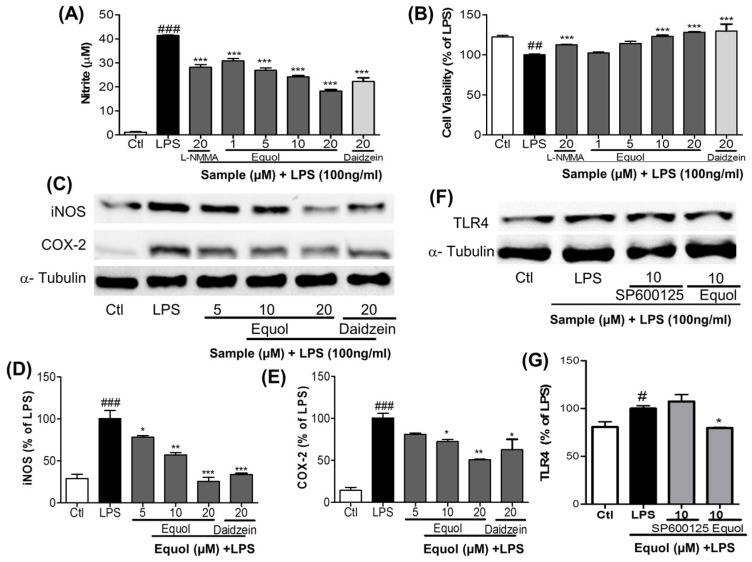
Effect of Equol on NO production, cell viability, and expression of iNOS, COX-2 and TLR4 expression in LPS-stimulated BV-2 cells. BV-2 cells were pretreated with various concentrations of Equol (μM) for 30 min, followed by treatment with LPS (100 ng/mL) for an additional 24 h to measure NO and MTT. 6 h treatment and LPS activation was used to measure iNOS and COX-2 expression via western blot analysis. (**A**) NO production measurement in LPS stimulated BV-2 cells. NMMA (20 μM) was used as a positive control; (**B**) Cell viability of BV-2 microglia following treatment with compounds with or without LPS; (**C**) Expression of iNOS and COX-2 in murine microglia; (**D**,**E**) Densitometric analysis of iNOS and COX-2 proteins; (**F**) Activation of TLR4 expression (**G**) Densitometric analysis for TLR4. α-Tubulin was used as the loading control. All data are presented as the mean ± SEM of three independent experiments. * *p* < 0.05, ** *p* < 0.01, *** *p* < 0.001 vs. LPS-treated group and ^#^ *p* < 0.05, ^##^ *p* < 0.01, ^###^ *p* < 0.001 vs. untreated control group.

Among various proinflammatory factors, NO is a harmful product released from activated microglia. As LPS is a TLR4 agonist, it activates this receptor and then initiates the inflammatory cascades in the microglial cells. Pretreatment of cells with Equol effectively decreased the LPS-induced TLR4 activation (Figure 2F,G) as well as NO production in BV-2 murine microglia cells without cellular toxicity (Figure 2A,B). A well-known inducible nitric oxide synthase inhibitor, NMMA [39], has an IC50 value of 24.35 µM, whereas Equol displayed an IC50 value of 3.00 µM for inhibition of NO production by LPS activated BV-2 cells (Figure 2A). This signified that the inhibitory effect of Equol was more potent than that of NMMA at a dose of 10 µM. Besides reducing NO production induced by LPS, Equol increased the viability of cells against LPS-induced cell death, suggesting a neuroprotective effect in LPS-mediated neuroinflammatory conditions. Additionally, Equol reduced the expression of iNOS and COX-2, as shown in Figure 2C–E. Equol inhibited the expression of iNOS and COX-2 by 78.12% ± 2.09%, 57.06% ± 2.76%, 25.59% ± 4.79% and 81.10% ± 1.39%, 72.36% ± 2.60%, 50.88% ± 0.74% at the concentration 5, 10, 20 µM, respectively. The effect of Equol at 20 µM is better than that of Daidzein as shown in Figure 2C–E).NO and PGE-2 are catalyzed by iNOS and COX-2, which are key neuroinflammatory enzymes in the brain. Equol dose dependently reduced the expression of iNOS and COX-2 in BV-2 microglia, displaying an inhibitory effect on NO and PGE-2 production. These data suggested that Equol may have significant anti-inflammatory properties in LPS-stimulated BV-2 cells. As LPS is a TLR4 receptor agonist, we checked the role of Equol and SP610025 for TLR4 inactivation. SP610035 did not alter the receptor expression, whereas Equol reduced its expression to 79.63% ± 0.47%.

### 3.3. Effect of Equol on LPS-Induced MAPK Signaling in LPS-Stimulated BV-2 Cells

**Figure 3:**
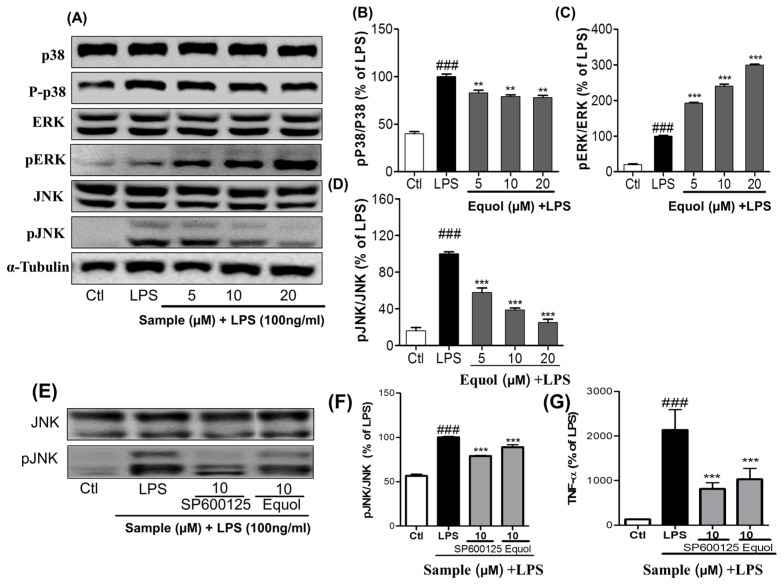
Effect of Equol on LPS-induced MAPK signaling in BV-2 cells. BV-2 cells were pretreated with or without Equol (μM) 30 min prior to LPS (100 ng/mL) stimulation. Activated cells were incubated for 30 min. (**A**) expression of pP38, P38, pJNK, JNK, and pERK, ERK; (**B**–**D**) Band intensities for pP38/P38, pERK/ERK, and pJNK/JNK as percentage of the LPS-treated group (set as 100%); (**E**) Phosphorylation of JNK to compare between Equol and SP600125 (**F**) Band intensities for pJNK/JNK (**G**) Secretion of TNF-α in 24 h LPS and compounds (Equol and SP600125) treated supernatant was measured using ELISA assay kit. SP600125 (JNK inhibitor) was used to compare the effect of Equol. All data are presented as the mean ± SEM of three independent experiments. ** *p* < 0.01, *** *p* < 0.001 vs. LPS-treated group and ^###^ *p* < 0.001 vs. untreated control group.

To further confirm the anti-inflammatory properties, we determined the effect of Equol on MAPK family modulation. In order to evaluate the effect of Equol on phosphorylation of p38, ERK, and JNK, we quantified their expressions via western blot analysis. Western blot analysis was carried out using the phosphorylated and total forms of antibodies against MAPKs (p38, ERK, and JNK). BV-2 cells were pretreated with different concentrations of Equol, followed by LPS stimulation for 30 min. Equol increased the phosphorylation of ERK, whereas the effect was opposite in the cases of p38 and JNK phosphorylation as shown in Figure 3A–D. Equol reduced the expression of phosphorylated p38 by 82.98% ± 2.67%, 79.10% ± 1.77% and 77.89% ± 2.32% at the concentration of 5, 10 and 20 µM respectively. Similarly, inhibition of JNK phosphorylation was 47.60% ± 5.00%, 38.87% ± 2.02% and 25.05% ± 3.80% at the same concentration of Equol. Unlikely, Equol concentration dependently increased the phosphorylation of ERK by 192% ± 2.17%, 240% ± 5.51% and 299.50% ± 2.57% at 5, 10 and 20 µM respectively. We compared the effect of Equol with well-known JNK inhibitor SP610025 to inhibit the JNK phosphorylation and TNF-α secretion in LPS-activated BV2 cells. In the treatment of same concentration of Equol and JNK inhibitor, they inhibit the phosphorylation of JNK by 79.11% ± 0.45% and 89.04% ± 1.87% respectively. Similarly, in the same condition, Equol and SP610025 inhibit the TNF-α production by 48.20% ± 9.37% and 37.98% ± 5.51%. Equol showed similar efficacy to inhibit the phosphorylation of JNK and TNF-α secretion in comparison to that of SP610025 as shown in Figure 3E–G. These data suggested that Equol could modulate the MAPK signaling by, in particular, inhibiting the JNK phosphorylation.

### 3.4. Effect of Equol on LPS-Induced NF-κB Activation in Murine Microglia Cells

**Figure 4:**
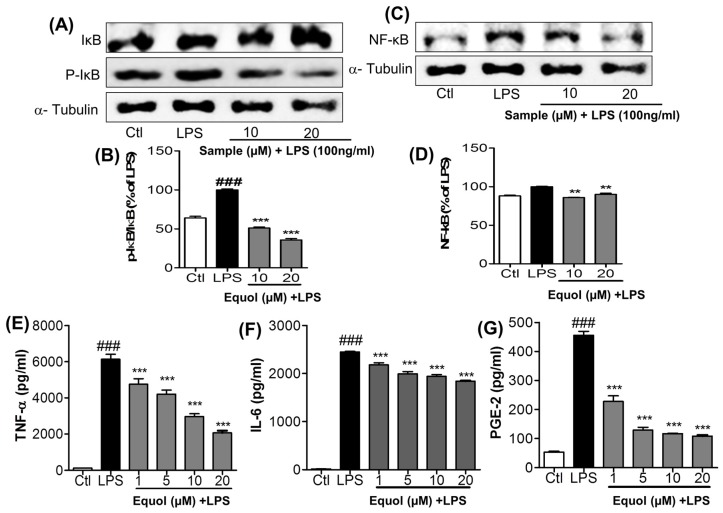
Effect of Equol on LPS-induced NF-κB activation and pro-inflammatory cytokines (TNF-α, IL-6) and PGE-2 secretion in BV-2 cells. BV-2 microglial cells were pretreated with 1, 5, 10, or 20 μM of Equol for 30 min and stimulated with LPS (100 ng/mL) for 1 h for NF-κB related proteins and 24 h for secreted cytokines measurement. Nuclear extracts were prepared using a nuclear extraction kit. Expression of NF-κB, IκB, and p-IκB were measured by western blot. (**A**,**B**) Protein levels of IκB and p-IκB and their band intensity, respectively; (**C**,**D**) NF-κB expression and its densitometric analysis. α-Tubulin was used as the loading control. The culture media was subsequently collected to measure the quantity of PGE-2, TNF-α, and IL-6 released by the cells; (**E**–**G**) secretion of TNF-α, IL-6 and PGE-2 in supernatant was measured using ELISA assay kit. Data are presented as mean ± SEM of three independent experiments performed in triplicate. ** *p* < 0.01, *** *p* < 0.001 vs. LPS-treated group and ^###^ *p* < 0.001 vs. untreated control group.

The effects of Equol on NF-κB activity were investigated using an NF-κB kit and western blot analysis as shown in Figure 4A–D.LPS significantly enhanced the expression of nuclear NF-κB in microglia. The increase in NF-κB activity was decreased significantly by pretreating cells with Equol at concentrations of 10 and 20 μM. It was reduced to 85.87% ± 0.45% and 90.02% ± 1.50% at those treated concentration. NF-κB is inactivated in the cytosol by binding to IκB and becomes active through translocation into the nucleus preceded by LPS-induced proteolytic degradation of IκB [37]. As shown in Figure 4A, IκB was phosphorylated and degraded 1h after LPS treatment. Pretreatment of BV-2 microglia cells with Equol (10 and 20 μM) decreased the phosphorylation and degradation of IκB in response to LPS, indicating that the subsequent NF-κB inactivation was induced by Equol as shown in Figure 4C,D.

### 3.5. Effect of Equol on TNF-α, IL-6, andPGE2 Production in LPS-Stimulated BV-2 Cells

Initiation of the inflammatory cascade produces many inflammatory mediators and proinflammatory cytokines, including TNF-α and IL-6. Production of proinflammatory cytokines further worsens the inflammatory cascade in neuroinflammatory diseases. Inhibition of the proinflammatory cytokines produced by activated microglia is necessary for neuroprotection. TNF-α was increased from 2.07% ± 0.01% over control to 100% ± 7.95% over control after 24 h of LPS treatment. It was then reduced to 77.48% ± 8.50%, 68.57% ± 6.17%, 48.29% ± 4.47%, and 33.63% ± 3.46% after Equol (1, 5, 10, and 20 μM) treatment with LPS respectively as shown in Figure 4E. Similarly, LPS-induced IL-6 production increased from 0.69 ± 0.068 to 100 ± 0.82 pg/mL after 24 h of LPS activation and Equol decreased its production concentration dependently and about 25% inhibition at 20 μM as shown in Figure 4F. PGE-2 from culture medium was increased from 53.12 ± 0.88 pg/mL in the control group to 455.6 ± 4.30 pg/mL after 24 h of exposure to LPS. PGE-2 synthesis was decreased to 227.52 ± 6.05 pg/mL, 128.78 ± 3.08 pg/mL, 116.66 ± 0.33 pg/mL, and 108.06 ± 1.16 pg/mL after treatment with Equol at concentrations of 1, 5, 10, and 20 μM, respectively as shown in Figure 4G.

Reduced proinflammatory cytokines, TNF-α and IL-6, as well as PGE-2production, in LPS activated BV-2 cells as shown in Figure 4E–G, further indicates the strong anti-inflammatory potential of Equol.

### 3.6. Effect of Equol on Activated Microglia-Induced Neurotoxicity in N2a Cells

**Figure 5:**
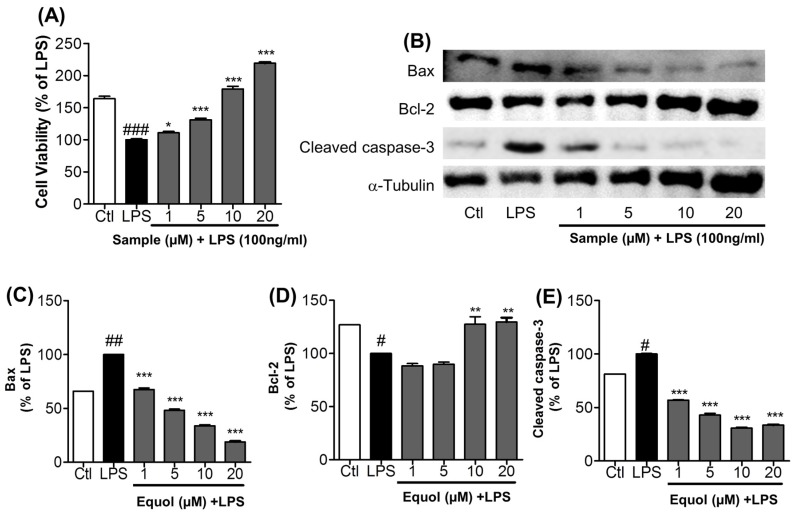
Effect of Equol on activated microglia-induced neurotoxicity in N2a cells. BV-2 microglial cells were pretreated with 1, 5, 10, or 20 μM of Equol for 30 min and stimulated with LPS (100 ng/mL) for 24 h. After 24 h of LPS treatment, the culture medium was collected and transferred to dishes plated with N2a cells. (**A**) Cell viability in N2a cells after 24 h treatment of LPS activated microglia treated media. Cell lysates were prepared in order to evaluate protein levels of apoptosis-related factors; (**B**) The expression of Bax, Bcl-2 and cleaved caspase-3 (**C**–**E**) Band intensity for above mentioned proteins respectively. α-Tubulin was used as the loading control. Data represent the mean ± SEM of three independent experiments performed in triplicate. * *p* < 0.05, ** *p* < 0.01, *** *p* < 0.001 vs. LPS-treated group and ^#^ *p* < 0.05, ^##^ *p* < 0.01, ^###^ *p* < 0.001 vs. untreated control group.

BV-2 cells were treated with Equol and with or without LPS (100 ng/mL) for 24 h. After 24 h of treatment, the treated media was collected carefully and transferred to N2a cells for another 24 h. Cell viability in N2a cells was measured using an MTT assay. More N2a cells treated with Equol survived than those that were only treated with LPS. Cell viability was measured as percentage of LPS; Equol increased the cell viability by 110.88% ± 4.94%, 131.03% ± 5.28%, 178.89% ± 9.49%, and 219.13% ± 3.97% at the concentrations of 1, 5, 10, and 20 μM, respectively as shown in Figure 5A. We next determined the effect of Equol on apoptosis in neuronal cells. First, Equol displayed a protective effect on LPS-activated BV-2 cells, mediated N2a toxicity as shown by MTT assay, and also induced toxicity via the expression of pro- and anti-apoptotic proteins in LPS-stimulated BV-2 cells. Equol decreased the expression of Bax and cleaved caspase-3 in a dose dependent manner. LPS treated group was made as 100% against untreated control. Equol treated conditioned medium dramatically reduced the expression of Bax by 67.45% ± 1.46%, 48.16% ± 1.23%, 33.84% ± 1.04% and 18.82% ± 0.83% at the concentration of 1, 5, 10 and 20 µM. Similarly, the treatment reduced the expression of cleaved caspase-3 by 56.87% ± 0.38%, 42.82% ± 1.64%, 30.76% ± 0.69% and 33.44% ± 0.55%. This value is lower than the untreated control too. Furthermore, Equol upregulates the expression of an anti-apoptotic protein Bcl2 from 100.0% ± 1.01% in LPS to 127.40% ± 7.20% and 129.60% ± 4.06% with the treatment of 10 and 20 µM as shown in Figure 5B–E. The dramatic change in the expression of pro-apoptotic and anti-apoptotic protein with the treatment of Equol, proved its neuroprotective effect. These results clearly suggest the neuroprotective effects of Equol in LPS-activated neuroinflammatory conditions.

### 3.7. Effect of Equol on Neurite Outgrowth in N2a Cells

**Figure 6:**
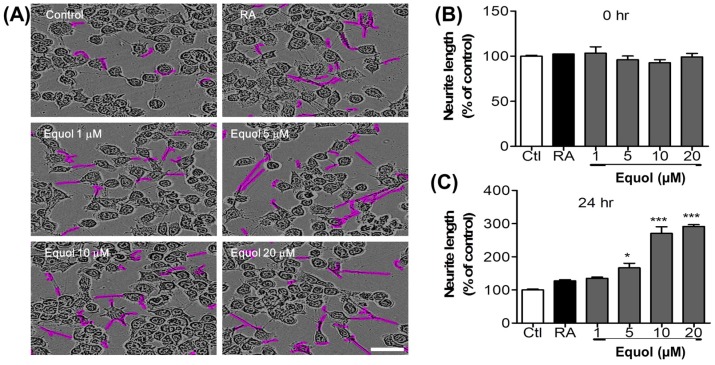

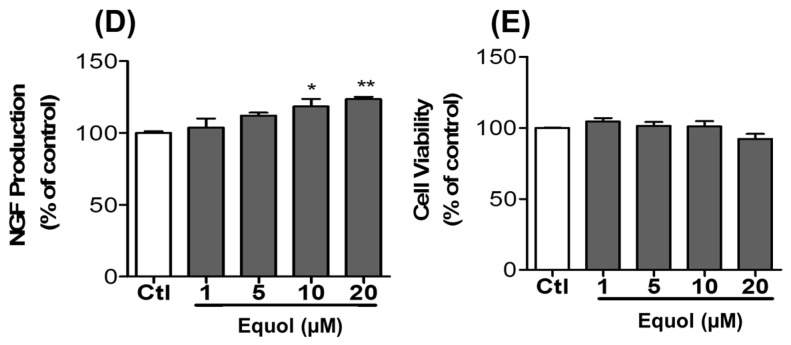
Effect of Equol on neurite outgrowth production in N2a cells and NGF production in C6 cells for neuroprotection. Neurite length in N2a cells was measured at regular intervals over a time span of 24 h after Equol (1, 5, 10, and 20 μM) treatment and images of cells were taken at the end of 24 h. (**A**) Neuronal cell morphology during treatment; Neurite outgrowth is shown in pink (scale bar = 50 μM) (**B**) Neurite length prior to treatment; (**C**) Neurite length after 24 h of treatment. Retinoic acid (10 μM) treatment was used as a positive control to stimulate neurite outgrowth. Retinoic Acid (RA) was used as a positive control. C6 cells were treated with Equol at concentrations of 1, 5, 10, and 20 μM, respectively. After 24 h, the amount of NGF produced by C6 cells was measured by ELISA; (**D**) NGF production in C6 glioma cells; (**E**) Cell viability of C6 cells during NGF production after 24 h of compound treatment. The data shown represent the mean ± SEM of three independent experiments performed in triplicate. * *p* < 0.05, ** *p* < 0.01, *** *p* < 0.001 vs. untreated control group.

N2a cells were treated with different concentrations of Equol and retinoic acid, and neurite outgrowth was evaluated. N2a cells cease to proliferate and begin to differentiate, as shown by neurite outgrowth, in response to serum starvation, retinoic acid, or growth factors such as neurotrophins and glial cell-derived neurotrophic factor family ligands, as shown in Figure 6A. Neurite length was measured using an IncuCyte imaging system until 24 h after treatment. Equol dose-dependently increased neurite length compared with untreated cells as shown in Figure 6B,C.

### 3.8. Effect of Equol on NGF Production in C6 Cells

In the neuronal defense system, once an antigen or inflammatory pathogen triggers the immune system, activated microglia produce a variety of inflammatory mediators to protect the brain from the pathogen. Among cells that protect the brain, astrocytes produce inflammatory cytokines as well as nerve growth factors to protect neuronal cells and to maintain homeostasis in the brain. Therefore, a compound that increases the production of NGF by activating astrocytes may also possess potential for neuroprotection. To measure NGF content in the medium, C6 cells were seeded onto 24 well plates and treated with different concentrations of Equol. NGF release in the medium was determined using an ELISA kit to confirm NGF protein expression in cells. Equol concentration dependently increased NGF levels to 103.81% ± 10.73%, 112.11% ± 3.46%, 118.48% ± 8.85%, and 123.5% ± 2.82% at concentrations of 1, 5, 10, and 20 μM, respectively, in a medium without cellular toxicity as shown in Figure 6D,E respectively.

## 4. Discussion

Estrogen shows neuroprotective effects by enhancing glia-neuron crosstalk [28]. Neuronal cell death caused by oxidative stress contributes to the pathogenesis of neurodegenerative diseases, such as Alzheimer’s disease and Parkinson’s disease. Because they contain high amounts of phenolic compounds, unsaturated oils and proteins, soybeans are a food with high nutritional value as well as antioxidant and anticancer properties due to their active metabolites [9]. Previous studies reported that soybean isoflavones may also have neuroprotective effects due to their strong antioxidant and anti-inflammatory activities [40]. Neuroprotective effects of phytoestrogens, especially Equol, were reported in cerebral ischemia, but the detailed mechanisms are not yet clear [41,42]. Additionally, Genistein was reported to have higher antioxidant activity than Daidzein because it has the same B-ring structure, but also has one more hydroxyl group on its A/C-ring. Because of this, Daidzein is more lipophilic than Genistein, which could compensate for Daidzein’s lower radical quenching capacity. Additionally, a Daidzein gut metabolite, Equol, is more soluble in organic solvents than Daidzein, meaning the bioavailability of Equol may be higher than that of Daidzein. Equol may be better in many aspects of biological activity than Genistein and Daidzein [43]. To find an effective therapeutic agent for the treatment of neurodegenerative diseases, including Alzheimer’s disease, it is essential to consider structures with good blood brain barrier (BBB)-permeability. Considering its chemical structure, Equol may have the better BBB-permeability than other phytoestrogen [11,44]. Therefore, in the present study, we reported the neuroprotective effect of Equol, a nonsteroidal estrogen produced by human metabolism, by targeting inhibition of LPS-induced microglial activation, neuroinflammation, and neuronal apoptosis.

Microglial cells that are part of the brain’s defense system normally respond to neuronal damage and induce phagocytosis [45,46]. However, toxin-induced chronic activation of microglia can cause neuroinflammation, followed by neurodegeneration and neuronal apoptosis in the brain, by inducing the production of proinflammatory cytokines (TNF-α and IL-6) and other inflammatory mediators, including reactive oxygen species (ROS), NO, MAPKs (phosphorylation of ERK, JNK, and p38), iNOS, COX-2, PGE-2, and glutamate [47,48,49]. Previous reports suggested that microglia-stimulated iNOS activation was the primary source of NO in neuroinflammation [50]. Among various proinflammatory factors, NO is a significant harmful product released from activated microglia. As LPS is a TLR4 agonist, it activates this receptor and then initiates the cascade of inflammation in the microglial cells. In the first screening of the Daidzein derivative for NO production inhibition, Equol showed the most potent activity without cellular toxicity (Figure 1A,B). The IC50 value of Equol is lower than that of Genistein and Daidzein and positive control L-NMMA. Not only is the Equol the most potent among Daidzein derivative, but it is also more promising than the well-known positive control L-NMMA (Figure 1C). Similarly, pretreatment of cells with Equol effectively decreased the LPS-induced TLR4 activation (Figure 2F,G) as well as NO production in BV-2 murine microglia cells without cellular toxicity (Figure 2A,B). In our study, Genistein, Daidzein and Equol all displayed inhibition of NO production in LPS-induced microglia, but Genistein was more toxic to neuronal cells than Daidzein. Equol was found to have the most potential and was without cellular toxicity. Additionally, Equol remarkably decreased iNOS and COX-2 expression, together with TNF-α and IL-6 secretion, in LPS-activated microglia in a concentration dependent manner. Because MAPKs respond to LPS-mediated microglia activation and mediate proliferation, differentiation and cell survival [51], short-term ERK1/2 activation promotes neuronal growth, differentiation and survival [52]. These signaling pathways and phosphorylation directly affect the NF-κB mediated transcription of these inflammatory mediators [53]. Equol increased the phosphorylation of ERK. However, it decreased the phosphorylation of p38 and JNK. Equol showed the similar efficacy to inhibit the phosphorylation of JNK and TNF-α secretion in comparison with a well-known JNK inhibitor (SP600125). NF-κB is a very important inflammatory mediator, and a modulator of neuroinflammation for the proinflammatory cytokines, iNOS and COX-2, in LPS-activated microglial cells [37]. Therefore, we evaluated the effect ability of Equol in altering the activity of NF-κB for the production of a variety of inflammatory mediators and pro-inflammatory cytokines that are responsible for neuroinflammation and neurodegeneration. Equol significantly inhibited NF-κB mediated transcription of proinflammatory mediators via decreased phosphorylation of IκB with the pretreatment of BV-2 cells. These results indicated that Equol had strong anti-inflammatory potential in LPS-activated microglia via reduced TLR4 activation, JNK phosphorylation and proinflammatory cytokine, especially TNF-α secretion. As TLR4-JNK-NF-κB-TNF-α mediated inflammatory cascades are the well-known pathway for the activated microglia-induced neuronal death, we moved to find the role of Equol to prevent LPS-activated microglia-induced neuronal cell death.

The activated microglia directly damage the neurons and induce neuronal apoptosis through the regulation of inflammatory and cytotoxic factors, including NO, TNF-α, IL-1β, arachidonic acid, eicosanoids and ROS. These cytotoxic factors are the major cause of neuronal damage and neuronal apoptosis through regulation of the Bcl-2 family [54,55,56]. The Bcl-2 family consists of pro-apoptotic (Bax and cleaved caspase-3) and pro-survival (Bcl-2) proteins, which are responsible for the physical and functional interactions that regulate apoptosis [57]. This study also supported the idea that LPS-activated microglia increased the expression of Bax and cleaved caspase-3 and downregulated Bcl-2 in neuronal cells, where Equol dramatically reversed the apoptotic properties of LPS-activated microglia cells and thereby protein expression in neuronal cells. Additionally, in support of an anti-apoptotic effect, Equol increased neuronal cell viability in LPS-activated microglia-induced neuronal death. These results suggested that Equol is a potent neuroprotective agent against LPS-stimulated neuronal death. Production of NO and increased COX-2, iNOS, PGE-2 and proinflammatory cytokines ultimately caused neuronal death. Neuronal death is mediated by increased pro-apoptotic proteins, such as Bax and cleaved caspase-3, and decreased pro-survival/anti-apoptotic proteins, such as Bcl-2.

Neuronal apoptosis is followed by high neurite loss after activated microglia-induced toxicity. This is one of the hallmarks of neuronal injury and neurodegeneration [58]. The neuronal immune system tried to adopt the neurite outgrowth and improve the functional recovery that is necessary for neuronal survival [59]. Apart from neuroprotection, reorganization of the lost neuronal network in the injured brain is necessary for the restoration of normal physiological function [60]. Natural compounds that restore neurite loss and promote neurite outgrowth may be considered potential neuroprotective agents [60]. In this study, Equol significantly increased neurite length and density relative to retinoic acid, demonstrating the strong neuroprotective efficacy of Equol in N2a cells. On the other hand, NGF, secreted by astrocytes, can repair inflammation-induced damage to neurons and prevent apoptotic neuronal cell death [61]. In this study, Equol substantially increased the secretion of NGF in C6 glioma cells, suggesting a neuroprotective effect.

## 5. Conclusions

In summary, Equol, a Daidzein gut metabolite, showed neuroprotective activity via downregulation of the LPS-induced neuroinflammation. It not only inhibited LPS-induced microglial activation, but also protected from neuroinflammatory injury through downregulation of neuronal apoptosis. Furthermore, it increased neurite outgrowth in neuronal cells and increased nerve growth in astrocytes. These results suggest that Equol may be a potential nutraceutical for neuroprotection via regulation of neuroinflammation, apoptosis, and neurotrophins.
